# Whole muscle 18F-choline uptake due to intense physical exercise

**DOI:** 10.1590/S1677-5538.IBJU.2018.0573

**Published:** 2019-09-02

**Authors:** Francisco Javier García-Gómez, Pablo Antonio de la Riva-Pérez, Ana Agudo-Martínez, Gertrudis Sabatel-Hernández, María Cinta Calvo-Morón

**Affiliations:** 1 Department of Nuclear Medicine, Virgen Macarena University Hospital,Seville, Spain

## INTRODUCTION

**Figure 1:**
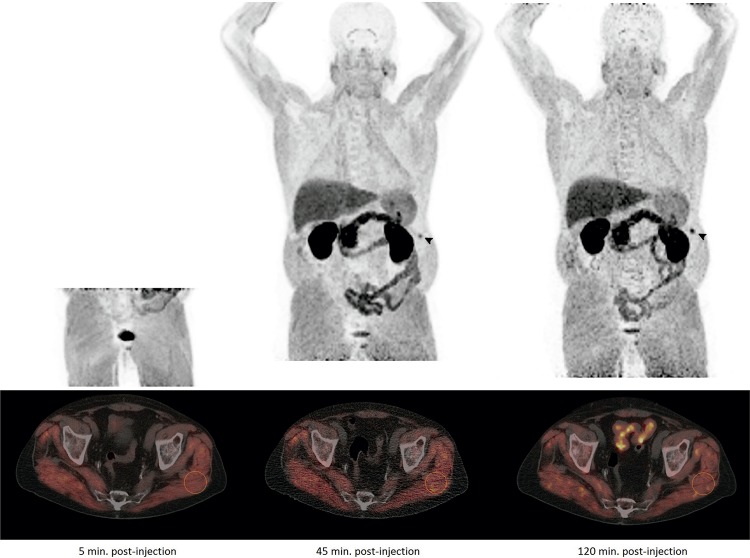
MIP and fused PET/CT images acquired at 5 min, 45 min and 120 min after intravenous injection of 18F-Choline. An intense and difuse uptake of radiotracer was observed in all the series of the study. The muscle activity was measured by circular ROI in gluteus maximus muscle (axial fused images) demonstrating stable activity throughout the exploration. Incidentally, a fracture of the eleventh left rib arch was also observed (head-arrow).

We report an 18F-choline PET/CT scan performed on a 74-year-old patient with history of prostate neoplasm in the clinical context of progressive PSA elevation (10ng/ml) with negative imaging tests. Pelvic images were acquired at 5 min. post-injection of standardized radiotracer dose, while whole-body images were obtained 45 and 120 min. after injection (Figure-1; MIP images). A homogeneous increased uptake of tracer in the whole axial and appendicular muscle structures was highlighted in all series. A SUVmax of 4.8, 5.0 and 4.9 were reached in a reference ROI at left gluteus maximus muscle (Figure-1; axial fused images). Additionally, a fracture of the eleventh left rib arch was also observed (head-arrow). An intense basal physical activity mainly based on long walks, farming tasks and dance classes were referred when the patient was re-interrogated. Taking medication that might interfere with tracer distribution, such as colchicine (1), was also ruled out. It is well known that 18F-choline can be uptake in different tissues including striated muscle (2, 3), although it is a poor referred finding which seems to be related to physical activity, being most likely due to increased local perfusion and probably unavoidable to some extent (3). Knowledge of these variability of physiological uptake, benign findings and pitfalls, is crucial in order to get the most out of the scan.
